# Preoperative Malignancy Risk Stratification in Bethesda IV Thyroid Nodules Using Integrated Clinical, Ultrasonographic, and Biochemical Parameters in Surgical Oncology Practice

**DOI:** 10.3390/diagnostics16193136

**Published:** 2026-09-27

**Authors:** Yavuz Selim Kahraman, İsmail Özer, Muhammet Raşit Aydın, Emre Gönüllü, Fuldem Mutlu, Adem Şentürk

**Affiliations:** 1 Department of General Surgery, Division of Surgical Oncology, Kastamonu Training and Research Hospital, Kastamonu 37150, Türkiye; 2 Department of Radiology, Sakarya University Faculty of Medicine, Sakarya 54290, Türkiye; ozer815@gmail.com (İ.Ö.); mutlufuldem@gmail.com (F.M.); 3 Department of Medical Education, Sakarya University Faculty of Medicine, Sakarya 54290, Türkiye; mraydin@sakarya.edu.tr; 4 Department of Gastroenterological Surgery, Sakarya University Training and Research Hospital, Sakarya 54100, Türkiye; egonullu@sakarya.edu.tr; 5 Department of Surgical Oncology, Sakarya Training and Research Hospital, Sakarya 54100, Türkiye; dr.adem.senturk@gmail.com

**Keywords:** thyroid nodule, fine-needle biopsy, thyroid neoplasms, ultrasonography, risk assessment

## Abstract

**Background and Objectives:** Bethesda Category IV thyroid nodules present a diagnostic challenge due to their variable risk of malignancy and uncertain cytological features. Therefore, determining the risk of malignancy before surgery is important. This study aims to identify clinical, biochemical, and ultrasonographic factors associated with malignancy and to explore the performance of a multivariable analysis based on routinely available preoperative features in thyroid nodules. **Methods:** This retrospective, single-center study included 105 patients who underwent thyroidectomy for Bethesda Category IV cytology. The mean age was 50.13 ± 11.78 years. 87 patients (82.9%) were female, and 18 (17.1%) were male. Patients were classified into benign/borderline (n = 79) and malignant (n = 26) groups based on their postoperative histopathological findings. Demographic characteristics, serum thyroid function tests, thyroid autoantibody levels, and ultrasonographic findings were compared. Variables significantly associated with malignancy were included in a Firth bias-reduced penalized multivariable logistic regression analysis. The discriminative performance of the multivariable analysis was evaluated using ROC curve analysis, and internal validation was performed using bootstrap resampling. The AUC was compared with that of ACR TI-RADS using DeLong’s test. **Results:** The malignant group showed a significantly higher prevalence of male sex, irregular margins, a taller-than-wide shape, and microcalcification (all *p* < 0.05). In the multivariable logistic regression analysis, only irregular margins (OR = 9.46, 95% CI: 1.88–47.59; *p* = 0.006) were independently associated with malignancy. The model demonstrated acceptable discrimination in this study population (n = 105), with an AUC of 0.717. The AUC of the multivariable analysis was 0.717, compared with 0.704 for ACR TI-RADS, and the difference was not statistically significant (ΔAUC = 0.013; DeLong *p* = 0.803). **Conclusions:** In Bethesda Category IV thyroid nodules, irregular margins remained independently associated with malignancy. The multivariable analysis did not demonstrate significantly better discrimination than ACR TI-RADS. These findings should be considered exploratory and require external validation in larger cohorts.

## 1. Introduction

Thyroid nodules are among the most common endocrine disorders observed in clinical practice. The detection rate has increased considerably with the widespread use of ultrasonography and cross-sectional imaging modalities. Although the vast majority of thyroid nodules are benign, the principal clinical challenge is the accurate identification of malignant nodules requiring oncologic treatment. Accurate preoperative assessment of malignancy risk is also important for clinical evaluation and patient counseling [1,2].

Fine-needle aspiration biopsy (FNAB) is one of the primary diagnostic methods used in the preoperative evaluation of thyroid nodules. The Bethesda System for Reporting Thyroid Cytopathology (TBSRTC) provides a standardized reporting system by classifying thyroid cytopathology into six diagnostic categories, each associated with a specific risk of malignancy and recommended clinical management. The latest edition includes important updates regarding TBSRTC terminology, estimates of malignancy risk, molecular testing, and clinical and radiological correlation. This system has aligned cytological reporting with the current pathological approaches described in the 2022 World Health Organization classification of thyroid neoplasms [2,3,4].

Bethesda Category IV, defined as suspicious for a follicular neoplasm, is one of the most challenging categories in the clinical management of thyroid cytopathology. The main reason for this is the biological and diagnostic characteristics of thyroid lesions exhibiting a follicular pattern. Bethesda Category IV alone is limited in reliably distinguishing benign from malignant nodules. Definitive diagnosis requires diagnostic surgery in most cases [2,3,5]. Although previous studies have shown that Bethesda Category IV nodules carry an intermediate risk of malignancy, more recent studies have demonstrated that malignancy rates may differ substantially from those predicted by the Bethesda system [5,6].

Thyroid lobectomy can be considered an appropriate initial option that is both diagnostic and therapeutic for many cytologically indeterminate nodules. However, total thyroidectomy may be preferred in the presence of bilateral disease, large nodules, suspicious ultrasonographic findings, a history of neck irradiation, a strong family history, or patient-specific clinical characteristics. Therefore, preoperative assessment of malignancy risk is one of several factors considered in individualized surgical planning [1,7,8]. From the perspective of surgical oncology, the primary challenge is not only determining the need for surgery. The key issue is to determine the most appropriate surgical approach for each patient. Inadequate surgical treatment may necessitate completion thyroidectomy if malignancy is confirmed following diagnostic lobectomy. Similarly, unnecessarily extensive surgery increases the risk of complications, such as hypoparathyroidism, recurrent laryngeal nerve injury, and the need for lifelong thyroid hormone replacement therapy in patients with benign disease. Therefore, preoperative assessment of malignancy risk may provide additional information in the clinical evaluation of patients with Bethesda Category IV nodules [7,8].

In recent years, studies have shown that selected ultrasonographic features, when evaluated together with cytological and clinical findings, may improve the prediction of malignancy and make a significant contribution to surgical decision-making [9,10]. Although large nodules are generally considered to carry a higher risk of malignancy in clinical practice, studies have shown that a nodule size threshold alone is not sufficiently reliable for determining the extent of surgery [5,11].

Molecular testing can provide a more accurate assessment of the risk of malignancy in cytologically indeterminate thyroid nodules. However, these tests are not yet part of routine clinical practice because of their high cost, limited availability across centers, and variable diagnostic performance in different populations. Therefore, in many healthcare systems and in routine surgical oncology practice, surgical planning is still largely based on readily available clinical, biochemical, cytological, and ultrasonographic data [1,6].

Accordingly, there remains a need for studies evaluating practical, reproducible, and readily applicable preoperative predictors of malignancy in Bethesda Category IV nodules. Such studies are of great importance, particularly because institution-specific malignancy rates and the availability of current diagnostic modalities directly influence surgical strategy. The study aimed to determine the malignancy rate among surgically treated patients with Bethesda Category IV thyroid nodules and to evaluate clinical, biochemical, and ultrasonographic factors associated with final histopathological malignancy. In addition, we explored the discriminative performance of a multivariable analysis based on routinely available preoperative features.

## 2. Materials and Methods

### 2.1. Study Design and Setting

The study was approved by the Sakarya University Non-Interventional Clinical Research Ethics Committee (Approval No: 150; Date: 24 May 2024). The study was conducted in accordance with the principles of the Declaration of Helsinki. This retrospective observational study was reported in accordance with the STROBE (Strengthening the Reporting of Observational Studies in Epidemiology) guidelines. The study aimed to generate preliminary evidence regarding clinical, biochemical, and ultrasonographic factors associated with malignancy in Bethesda Category IV thyroid nodules.

### 2.2. Study Population and Setting

Patients who underwent ultrasound-guided fine-needle aspiration biopsy (FNAB) were included in the study. Among these patients, those with a Bethesda Category IV cytology report who subsequently underwent thyroidectomy were included in the study. Patients without a histopathological diagnosis, those with incomplete clinical or laboratory data, those with a history of previous thyroid surgery, those who underwent surgery for recurrent thyroid malignancy, or those whose preoperative cytology was reported as a category other than Bethesda Category IV were excluded from the study. All cases were evaluated according to the consecutive patient principle.

### 2.3. Inclusion Criteria and Exclusion Criteria

A retrospective review of the records from the study period identified a total of 680 patients who underwent thyroidectomy. All patients underwent preoperative fine-needle aspiration biopsy (FNAB), and 137 (20.1%) were diagnosed cytologically as Bethesda Category IV [Follicular Neoplasm/Suspicious for a Follicular Neoplasm (FN/SFN)]. After applying the predefined exclusion criteria, 21 patients with incomplete laboratory data, 7 patients with incomplete ultrasonographic evaluation, 3 patients with a history of previous thyroid surgery, and 1 patient with missing histopathological data were excluded from the study. As a result, a total of 105 patients were included in the study. The patient selection flow diagram is presented in Figure 1.

### 2.4. Data Collection

The study was conducted through a retrospective review of the medical records of patients who underwent surgical treatment for a cytological diagnosis of Bethesda Category IV at the Surgical Oncology Clinic between January 2020 and April 2024. Cytological evaluations were reported according to the Bethesda System. Only cases classified as Bethesda Category IV were included in the study. Definitive postoperative histopathological diagnoses were obtained from the pathology reports. Clinical, laboratory, radiological, cytological, and histopathological data were retrospectively obtained from the electronic medical record system and pathology archives. After patient selection, demographic, clinical, and laboratory variables were recorded during data collection. In addition, preoperative ultrasonographic findings and definitive postoperative histopathological diagnoses were recorded. Preoperative ultrasonographic findings included data on nodule composition, echogenicity, shape, margin, and echogenic foci obtained from routine ultrasonography reports. ACR TI-RADS categories documented in the original preoperative ultrasonography reports were also recorded. For the purpose of this study, each ultrasonographic feature was analyzed separately to evaluate its association with histopathological malignancy. Operative data were also retrieved from the institutional surgical records, including the type of initial thyroid procedure and whether subsequent completion thyroidectomy was performed during the available follow-up. The primary endpoint of the study was the presence of malignancy on postoperative histopathological examination. The benign group included patients with follicular adenoma, nodular hyperplasia, Hashimoto thyroiditis, and other benign lesions. The malignant group included patients with papillary thyroid carcinoma, follicular thyroid carcinoma, Hürthle cell carcinoma, and other malignant tumors. NIFTP cases were not included in the malignant group and were classified as benign/borderline lesions. NIFTP was classified as a borderline lesion according to current WHO recommendations.

### 2.5. Statistical Analyses

Statistical analyses were performed using IBM SPSS Statistics for Windows, Version 30.0 (IBM Corp., Armonk, NY, USA). Normality of distribution was assessed using the Shapiro-Wilk test. Continuous variables were compared between two groups using the independent samples *t*-test or the Mann-Whitney U test. Categorical variables were compared using the Pearson chi-square test or Fisher’s exact test, as appropriate. Variables significantly associated with malignancy in the univariable analysis were included in the multivariable analysis. Accordingly, male sex, microcalcifications, taller-than-wide shape, and irregular margins were included in a Firth bias-reduced penalized logistic regression model. The internal validity of the final multivariable analysis was assessed using 1000 bootstrap resamplings. The diagnostic performance of continuous variables in predicting malignancy was evaluated using receiver operating characteristic (ROC) curve analysis. The area under the curve (AUC) was calculated along with the 95% confidence interval (CI). The AUC of the multivariable analysis was directly compared with that of ACR TI-RADS using DeLong’s test for correlated ROC curves, and the difference in AUC (ΔAUC) was calculated. An exploratory probability threshold was determined using the Youden index. This data-based threshold was used to define the sensitivity and specificity of the model in the current study population and was not considered a clinically validated decision threshold. Statistical significance was set at *p* < 0.05 in all analyses.

## 3. Results

### 3.1. Patient Selection and Baseline Characteristics

Bethesda Category IV cytology was detected in 137 of 680 patients. After excluding 32 patients, 105 patients were included in the final analysis, of whom 79 (75.2%) had benign/borderline lesions, and 26 (24.8%) had malignant lesions. The flow diagram regarding patient selection is presented in Figure 1.

Based on the histopathological characteristics, 79 patients (75.2%) were benign/borderline, and 26 patients (24.8%) were malignant. Papillary thyroid carcinoma (n = 18, 69.2%) was found to be the most frequent histological subtype among malignant cases. Among the 18 PTCs, 9 (50.0%) were follicular-patterned, 7 (38.9%) were classical/conventional PTC, and 2 (11.1%) represented other PTC subtypes. This was followed by follicular thyroid carcinoma (n = 4, 15.4%), Hürthle cell/oncocytic carcinoma (n = 2, 7.7%), and other malignant tumors (n = 2, 7.7%) (Table 1).

The mean age of the overall study population was 50.13 ± 11.78 years. The mean age was 51.10 ± 11.9 years in the benign/borderline group and 47.2 ± 11.10 years in the malignant group, with no significant difference between the groups (*p* = 0.132). Overall, 87 patients (82.9%) were female, and 18 (17.1%) were male. Although female sex predominated in both groups, the proportion of male patients was higher in the malignant group than in the benign/borderline group (30.8% vs. 12.7%); however, this difference was not statistically significant (*p* = 0.068). Although ultrasonographic findings suggestive of chronic autoimmune thyroiditis (Hashimoto thyroiditis) were more frequent in the benign/borderline group than in the malignant group (35.4% vs. 23.1%), no significant difference was observed between the groups (*p* = 0.354) (Table 2).

### 3.2. Preoperative Laboratory Characteristics

The preoperative laboratory parameters of the patients are presented in Table 3. Serum TSH [1.73 (0.80–2.86) mIU/L vs. 1.25 (0.57–1.77) mIU/L; *p* = 0.098], free T4 [13.12 (11.70–14.29) pmol/L vs. 12.58 (10.65–13.69) pmol/L; *p* = 0.272], and free T3 [4.16 (3.41–4.59) pg/mL vs. 4.25 (3.79–4.93) pg/mL; *p* = 0.199] levels did not differ significantly between the benign/borderline and malignant groups. Although anti-TPO levels were higher in the malignant group, the difference was not statistically significant (*p* = 0.228). Similarly, anti-Tg levels did not differ between the groups [2.38 (1.44–7.98) IU/mL vs. 1.40 (1.10–13.26) IU/mL; *p* = 0.302]. None of the preoperative biochemical parameters evaluated showed a significant association with malignancy in Bethesda Category IV thyroid nodules.

### 3.3. Preoperative Ultrasonographic Characteristics

The comparison of preoperative ultrasonographic characteristics according to the final histopathological diagnosis is presented in Table 4. The median nodule size was 20.0 mm (14.0–33.0) in the benign/borderline group and 24.0 mm (13.5–39.0) in the malignant group, with no significant difference between the groups (*p* = 0.458). No significant differences were observed between the groups with respect to ultrasonographic findings of chronic thyroiditis/heterogeneity (34.2% vs. 26.9%; *p* = 0.631), nodule composition (*p* = 0.294), or echogenicity (*p* = 0.666). In contrast, ultrasonographic features suspicious for malignancy were observed significantly more frequently in the malignant group. The prevalence of a taller-than-wide shape (26.9% vs. 3.8%; *p* = 0.002) and irregular margins (38.5% vs. 2.5%; *p* < 0.001) was significantly higher in the malignant group. The overall distribution of echogenic foci differed significantly between the benign/borderline and malignant groups (Fisher’s exact test, *p* = 0.002). Microcalcifications were observed in 6 of 26 (23.1%) malignant nodules compared with 1 of 79 (1.3%) benign/borderline nodules, whereas macrocalcifications were present in 3 of 26 (11.5%) and 16 of 79 (20.3%) nodules, respectively (Table 4).

Of the 105 patients, 80 (76.2%) underwent total thyroidectomy, and 25 (23.8%) underwent lobectomy as the initial surgical procedure. No patient underwent completion thyroidectomy. Malignant histopathology was identified in 22 of 80 patients who underwent total thyroidectomy and in 4 of 25 patients who underwent lobectomy, with no statistically significant difference between the groups (Fisher’s exact test, *p* = 0.298). Also, the median estimated malignancy probability was similar between patients who underwent total thyroidectomy and those who underwent lobectomy (0.145 vs. 0.145; Mann–Whitney U test, *p* = 0.358).

In an additional analysis, ACR TI-RADS categories were evaluated for the 105 Bethesda Category IV nodules. None of the nodules were classified as TR1; 10 (9.5%) were classified as TR2, 38 (36.2%) as TR3, 46 (43.8%) as TR4, and 11 (10.5%) as TR5. The malignancy rates were 10.0% (1/10), 15.8% (6/38), 21.7% (10/46), and 81.8% (9/11) for TR2, TR3, TR4, and TR5, respectively. A significant association was observed between ACR TI-RADS category and malignant histopathology (*p* < 0.001). When ACR TI-RADS category was analyzed as an ordinal predictor, each one-category increase was associated with higher odds of malignancy (OR = 3.22, 95% CI: 1.62–6.42; *p* < 0.001). Using TR5 as the high-risk threshold yielded a sensitivity of 34.6% and a specificity of 97.5% for malignant histopathology (Table 5).

### 3.4. Univariate and Multivariable Logistic Regression Analyses for Predictors of Malignant Histopathology

Age, Hashimoto thyroiditis, TSH level, nodule size, solid composition, and hypoechogenicity were not significantly associated with malignancy (all *p* > 0.05). In contrast, male sex (OR = 3.07, 95% CI: 1.06–8.89; *p* = 0.039), a taller-than-wide shape (OR = 9.33, 95% CI: 2.20–39.51; *p* = 0.002), irregular margins (OR = 24.06, 95% CI: 4.81–120.48; *p* < 0.001), and the presence of microcalcifications (OR = 23.40, 95% CI: 2.66–205.61; *p* = 0.004) were significantly associated with malignancy.

Given the limited number of malignant outcomes (n = 26), the revised multivariable analysis was performed using Firth bias-reduced penalized logistic regression with a fixed four-variable model including male sex, taller-than-wide shape, irregular margins, and presence of microcalcifications. Irregular margins were independently associated with malignant histopathology (aOR = 9.46, 95% CI: 1.88–47.59; *p* = 0.006) (Table 6).

### 3.5. ROC Analysis and Internal Validation of the Multivariable Analysis

The diagnostic performance of the multivariable prediction model for predicting malignant histopathology was evaluated using receiver operating characteristic (ROC) analysis in Table 7. In 105 patients, the fixed four-variable Firth model demonstrated an apparent AUC of 0.717 (95% CI: 0.603–0.831). The exploratory probability threshold was ≥0.461, corresponding to a sensitivity of 46.2% and a specificity of 94.9%. This data-derived threshold was considered exploratory and was not interpreted as a clinically validated decision threshold. Internal validation using 1000 bootstrap resamples yielded an estimated optimism of 0.016 and an optimism-corrected AUC of 0.701. For comparison, ACR TI-RADS yielded an AUC of 0.704. Direct comparison using DeLong’s test showed no statistically significant difference between the multivariable analysis and ACR TI-RADS (ΔAUC = 0.013; *p* = 0.803).

## 4. Discussion

In this study, preoperative factors predicting malignancy were evaluated in patients who underwent surgery for Bethesda Category IV (Follicular Neoplasm/Suspicious for a Follicular Neoplasm) cytology. Male sex, microcalcifications, a taller-than-wide shape, and irregular margins were significantly associated with malignancy in the univariable analyses. However, after multivariable adjustment, only irregular margins remained independently associated with malignant histopathology. The multivariable analysis showed an AUC of 0.717, with an optimism-corrected AUC of 0.701 after bootstrap internal validation. Importantly, its discriminative performance was not significantly different from that of ACR TI-RADS (AUC 0.704; ΔAUC = 0.013; DeLong *p* = 0.803). These findings suggest that irregular margins may provide relevant preoperative information in Bethesda Category IV nodules; however, the present findings should be considered exploratory rather than evidence of a clinically ready prediction tool [12,13,14].

Bethesda Category IV nodules represent one of the most challenging groups in the clinical management of thyroid cytology. The main challenge in this category is the inability to distinguish follicular adenoma from follicular thyroid carcinoma on cytology alone, as a definitive diagnosis requires the demonstration of capsular and/or vascular invasion. Therefore, surgery in this patient group often serves as both a diagnostic and a therapeutic procedure. Identifying readily available preoperative predictors associated with an increased likelihood of malignancy is important for oncology practice [13,15,16].

The malignancy rate observed in our study is consistent with that reported in recent surgical series of Bethesda Category IV nodules. The literature indicates that these rates vary considerably among centers. The main reasons for this variability include differences in surgical referral policies, cytopathological interpretation, patient population characteristics, the distribution of ultrasonographic risk features, and the use of molecular testing. Kuru and Kefeli reported that the factors associated with malignancy and referral for surgery in Bethesda Category IV nodules cannot be explained by cytology alone and that clinical and radiological parameters also play a role in risk stratification [13]. Similarly, it has been shown that patient characteristics, ultrasonographic findings, and molecular testing collectively influence the risk of malignancy in Bethesda Category III–V nodules [12]. These findings support the interpretation that the malignancy rate observed in our study should be evaluated in conjunction with the preoperative risk profile.

With respect to demographic variables, no statistically significant association between age and malignancy was observed in our study. The predictive value of age in Bethesda Category IV nodules is inconsistent in the literature. Although some studies have reported that younger or older age may be associated with different tumor biologies, age alone does not appear to be sufficiently reliable to determine the extent of surgery in patients with indeterminate cytology [12,17]. Serum TSH was not significantly associated with malignancy in the present study. Although previous studies have suggested that TSH may contribute to malignancy risk assessment in patients with indeterminate thyroid nodules [16], this association was not observed in our cohort. This discrepancy may be related to the relatively small sample size and the limited number of malignant cases in our study.

In our study, preoperative ultrasonographic findings suggestive of chronic autoimmune thyroiditis (Hashimoto thyroiditis) and serum thyroid autoantibody levels were not significantly associated with malignancy. Rago et al. reported that the clinical features of thyroid autoimmunity in patients with indeterminate cytology were associated with histological thyroiditis but were not reliable predictors of malignancy [17]. Our findings suggest that preoperative ultrasonographic features of chronic thyroiditis were not significantly associated with malignancy in Bethesda Category IV nodules. However, because thyroid autoantibody levels were not available for all patients, interpret findings for these variables with caution.

Irregular margins, a taller-than-wide shape, and microcalcifications were significantly associated with malignancy in the univariable analyses. However, after multivariable adjustment, only irregular margins remained independently associated with malignancy. Chng et al. demonstrated the value of sonographic features for malignancy risk stratification in nodules with follicular neoplasm cytology [14]. Similarly, Latia et al. reported associations between ultrasonographic and elastographic findings and histopathological outcomes in Bethesda Category IV nodules [15]. These studies support the relevance of ultrasonographic features in the assessment of cytologically indeterminate nodules [9,14,15].

Irregular margins remained independently associated with malignancy in the multivariable analysis. Irregular or infiltrative margins may reflect tumor growth at the interface with the surrounding thyroid parenchyma. Ultrasound-based risk-stratification systems, such as ACR TI-RADS and EU-TIRADS, also recognize irregular margins as a suspicious sonographic feature [18,19]. In the present study, irregular margins were predominantly observed in papillary thyroid carcinomas. Therefore, although irregular margins were independently associated with malignancy in the overall Bethesda Category IV cohort, our findings do not support a subtype-specific association with follicular-patterned malignancies.

A taller-than-wide shape was significantly associated with malignancy in the univariable analysis but did not remain statistically significant after multivariable adjustment. Previous studies have reported that ultrasonographic features, together with other clinical parameters, may contribute to malignancy risk stratification in indeterminate thyroid nodules [12]. In our cohort, we observed a taller-than-wide shape across both papillary and follicular-patterned malignant subtypes; however, the limited number of non-papillary malignant cases precludes subtype-specific conclusions.

Although microcalcifications were significantly associated with malignancy in the univariable analysis, they did not remain independently associated with malignancy after multivariable adjustment. Rosário et al. emphasized that readily available imaging modalities contribute to risk assessment in nodules with indeterminate cytology; however, no single imaging finding is sufficient to guide surgical decision-making [20]. Therefore, in Bethesda category IV nodules, microcalcifications appear to be more appropriately interpreted as part of a composite risk assessment rather than as an isolated predictor.

In our study, nodule size was not significantly associated with malignancy. Kiernan and Solórzano reported that nodule size has limited value in predicting malignancy in Bethesda III–V nodules and should not be considered a reliable criterion for surgical decision-making when used in isolation [21]. Our findings are consistent with this observation. These findings suggest that ultrasonographic morphological features may warrant further evaluation as candidate factors in future prospective risk-stratification studies.

In the present study, however, the discriminative performance of the multivariable analysis was not significantly different. Therefore, these findings do not demonstrate incremental discriminative performance beyond the established ACR TI-RADS classification. Previous studies have explored predictive approaches incorporating clinical and radiological variables for malignancy risk assessment in cytologically indeterminate thyroid nodules [22,23,24]. Given the small sample size, limited number of malignant cases, and lack of external validation, the present findings should be considered exploratory and hypothesis-generating rather than evidence of a clinically ready prediction tool.

Molecular testing is playing an increasingly important role in the management of cytologically indeterminate thyroid nodules. However, these tests are not universally available, and their cost-effectiveness varies across different healthcare systems. Payne et al. demonstrated that molecular testing may increase the likelihood of optimal surgical management in Bethesda III–IV thyroid nodules [25]. Chowdhury et al., in a systematic review and meta-analysis, highlighted that molecular testing has the potential to reduce unnecessary surgeries, although variations in diagnostic performance exist among different testing platforms [26]. Therefore, in centers where molecular testing is not routinely available, risk assessment based on clinical and ultrasonographic variables remains a practical and feasible approach for guiding clinical decision-making.

From a surgical oncology perspective, the relationship between retrospectively estimated malignancy risk and the actual initial surgical approach was additionally evaluated. Predicted malignancy probabilities did not differ significantly between patients who underwent total thyroidectomy and those who underwent lobectomy. Valderrabano and McIver emphasized that risk stratification in indeterminate thyroid nodules should extend beyond cytological diagnosis alone [27]. Similarly, Kuta et al. showed that treatment strategies for Bethesda Category III–IV nodules vary by patient-, surgeon-, and institution-related factors, underscoring the importance of patient-centered decision-making [28]. In the present study, the retrospectively estimated malignancy risk was not significantly associated with the actual initial surgical approach. Moreover, clinicians did not have access to these risk estimates at the time of surgery. Therefore, the present findings should not be interpreted as supporting their use to determine the extent of surgery, and any potential role in surgical planning requires prospective evaluation and external validation.

The strengths of this study include the evaluation of a homogeneous study population consisting exclusively of patients who underwent surgery for Bethesda Category IV cytology, the availability of definitive histopathological diagnoses for all cases, the integrated analysis of clinical, biochemical, and ultrasonographic variables, and the bootstrap-based internal assessment of the multivariable analysis. The study extends beyond merely reporting the malignancy rate and identifies candidate preoperative factors that may inform future risk-stratification studies.

The study has certain limitations. The retrospective, single-center design and relatively small sample size may limit the generalizability of the findings. In addition, because only surgically treated patients were included, selection bias is possible, and the observed malignancy rate may not represent all Bethesda Category IV nodules. In particular, the limited number of malignant cases reduced the precision of the multivariable estimates, as reflected by the wide confidence intervals. Although papillary thyroid carcinoma represented the majority of malignant cases, the numbers of follicular thyroid carcinoma and oncocytic (Hürthle cell) carcinoma were small, limiting subtype-specific analyses and the ability to draw conclusions regarding the classical follicular-patterned diagnostic challenge of Bethesda Category IV nodules. Molecular testing was not routinely available during the study period, which further limited contemporary assessment of cytologically indeterminate thyroid nodules. Although bootstrap-based internal validation was performed, external validation was not available. Therefore, the present findings should be considered exploratory and hypothesis-generating and require validation in larger, independent, multicenter cohorts before any potential clinical application.

## 5. Conclusions

In this study, irregular margins were independently associated with malignancy in Bethesda Category IV thyroid nodules. Although male sex, microcalcifications, and a taller-than-wide shape were associated with malignancy in the univariable analyses, these associations did not remain statistically significant after multivariable adjustment. The multivariable analysis did not demonstrate significantly better discrimination than ACR TI-RADS. From a surgical oncology perspective, retrospectively estimated malignancy risk was not significantly associated with the actual initial surgical approach. Therefore, the present findings should not be interpreted as supporting the use of this analysis to determine the extent of thyroid surgery. The findings should be considered exploratory and require external validation in larger, independent cohorts and prospective evaluation before any potential clinical application.

## Figures and Tables

**Figure 1:**
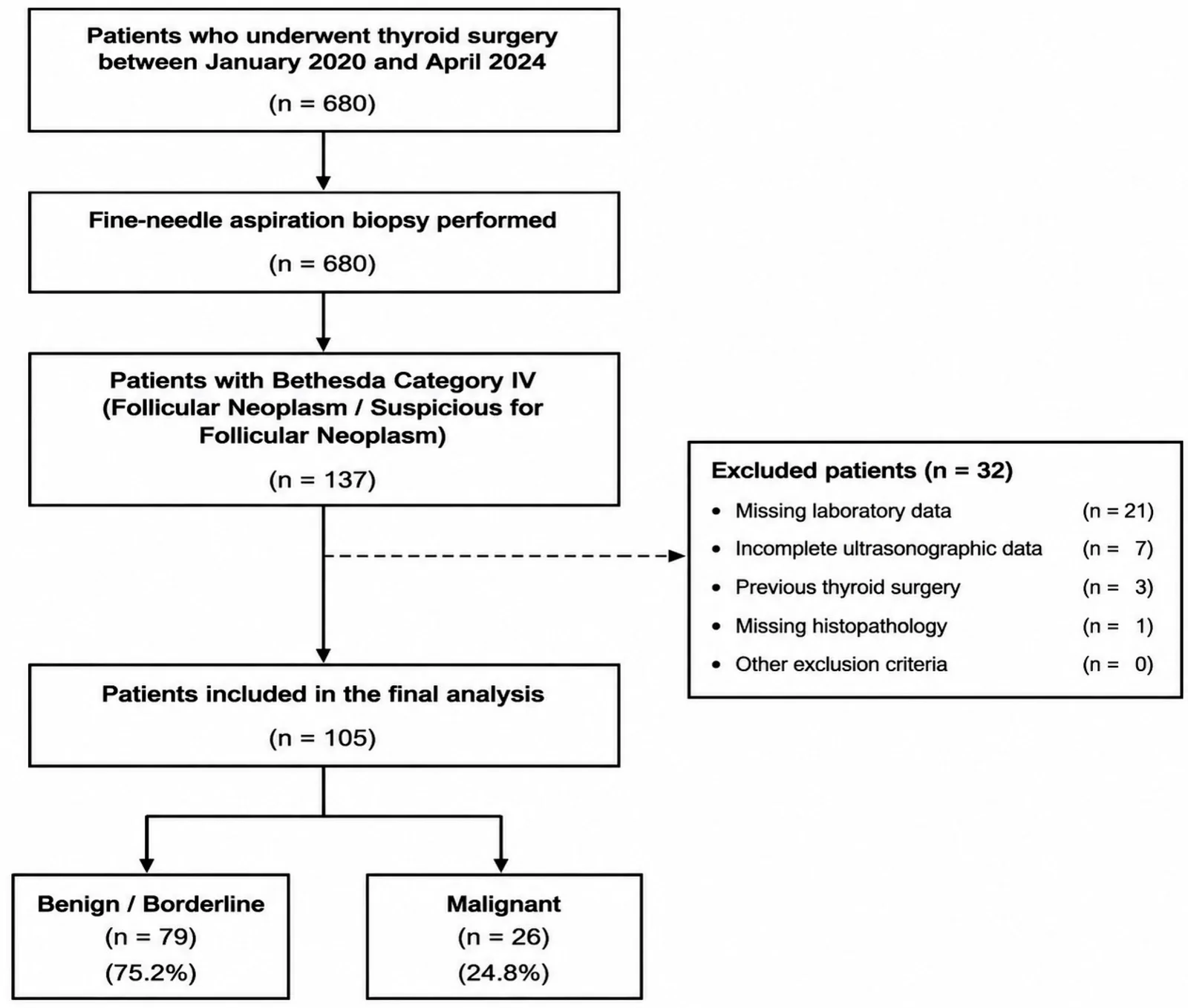
Study flow diagram of patient selection. From 680 consecutive patients who underwent thyroid surgery, 137 had Bethesda Category IV cytology. After applying exclusion criteria, 105 patients were included in the final analysis.

**Table 1:** Distribution of histopathological diagnosis characteristics of patients.

Histopathological Diagnoses	n	%
Benign		
Benign lesions	75	71.4
Borderline		
Borderline lesions (NIFTP)	4	3.8
Malignant		
Papillary thyroid carcinoma	18	17.1
Follicular thyroid carcinoma	4	3.8
Hürthle cell/oncocytic carcinoma	2	1.9
Other malignant tumors	2	1.9
Total	105	100.0

Abbreviations: NIFTP, non-invasive follicular thyroid neoplasm with papillary-like nuclear features; n, number; %, percent.

**Table 2:** Demographic and clinical characteristics of patients.

Patients Characteristics	Benign/Borderline (n = 79)	Malignant (n = 26)	Total (n = 105)	*p*-Value
Age (years) Mean ± SD	51.10 ± 11.9	47.2 ± 11.10	50.13 ± 11.78	0.132 ^1^
Female, n (%)	69 (87.3)	18 (69.2)	87 (82.9)	0.068 ^2^
Male, n (%)	10 (12.7)	8 (30.8)	18 (17.1)	
Preoperative ultrasonographic thyroiditis (Hashimoto features), n (%)	28 (35.4)	6 (23.1)	34 (32.4)	0.354 ^2^

Abbreviations: ^1^ Student’s *t*-test, ^2^ Pearson chi-square test. SD, standard deviation; n, number; %, percent.

**Table 3:** Comparison of preoperative laboratory parameters according to final histopathological diagnosis.

Laboratory Parameters	Benign/Borderline (n = 79)	Malignant (n = 26)	*p*-Value *
TSH (mIU/L)	1.73 (0.80–2.86)	1.25 (0.57–1.77)	0.098
FT4 (pmol/L)	13.12 (11.70–14.29)	12.58 (10.65–13.69)	0.272
FT3 (pg/mL)	4.16 (3.41–4.59)	4.25 (3.79–4.93)	0.199
Anti-TPO (IU/mL)	0.68 (0.14–73.10)	15.25 (0.51–111.13)	0.228
Anti-Tg (IU/mL)	2.38 (1.44–7.98)	1.40 (1.10–13.26)	0.302

* Mann–Whitney U test; Abbreviations: TSH, thyroid-stimulating hormone; FT4, free thyroxine; FT3, free triiodothyronine; Anti-TPO, anti-thyroid peroxidase antibody; Anti-Tg, anti-thyroglobulin antibody.

**Table 4:** Association between ultrasonographic features and surgical approach with final pathology in Bethesda IV thyroid nodules.

Variable	Benign/Borderline (n = 79)	Malignant (n = 26)	*p*-Value
Nodule size, mm	20.0 (14.0–33.0)	24.0 (13.5–39.0)	0.458 *
Chronic thyroiditis, n (%)	27 (34.2)	7 (26.9)	0.631 **
Composition, n (%)			
Spongiform	5 (6.3)	0 (0.0)	0.294 ***
Cystic	4 (5.1)	0 (0.0)	
Mixed cystic-solid	21 (26.6)	6 (23.1)	
Solid	49 (62.0)	20 (76.9)	
Echogenicity, n (%)			
Isoechoic	23 (29.1)	6 (23.1)	0.666 ***
Hyperechoic	9 (11.4)	2 (7.7)	
Hypoechoic	47 (59.5)	18 (69.2)	
Taller-than-wide, n (%)			
Absent	76 (96.2)	19 (73.1)	0.002 **
Present	3 (3.8)	7 (26.9)	
Margin, n (%)			
Regular	77 (97.5)	16 (61.5)	<0.001 **
Irregular	2 (2.5)	10 (38.5)	
Echogenic foci, n (%)			
Absent	62 (78.5)	17 (65.4)	0.002 **
Macrocalcification	16 (20.3)	3 (11.5)	
Microcalcification	1 (1.3)	6 (23.1)	
Initial surgical approach			
Bilateral total thyroidectomy	58 (73.4)	22 (84.6)	0.298 **
Unilateral lobectomy	21 (26.6)	4 (15.4)	

* Mann–Whitney U test; ** Fisher’s exact test; *** Pearson chi-square test.

**Table 5:** ACR TI-RADS categories according to final histopathology in Bethesda Category IV thyroid nodules.

ACR TI-RADS Category	Benign/Borderline (n = 79)	Malignant (n = 26)	Total (n = 105)	Malignancy Rate, %
TR1	0 (0.0)	0 (0.0)	0	—
TR2	9 (90.0)	1 (10.0)	10	10.0
TR3	32 (84.2)	6 (15.8)	38	15.8
TR4	36 (78.3)	10 (21.7)	46	21.7
TR5	2 (18.2)	9 (81.8)	11	81.8
Total	79 (75.2)	26 (24.8)	105	24.8

Abbreviations: ACR, American College of Radiology; TI-RADS, Thyroid Imaging Reporting and Data System; TR1, benign; TR2, not suspicious; TR3, mildly suspicious; TR4, moderately suspicious; TR5, highly suspicious.

**Table 6:** Univariable and multivariable logistic regression analyses for predictors of malignant histopathology.

Variable	Univariate OR (95% CI)	*p* Value	Adjusted OR (95% CI)	*p* Value
Age	0.97 (0.93–1.01)	0.146	–	–
Male sex	3.07 (1.06–8.89)	0.039	2.14 (0.62–7.40)	0.229
Hashimoto’s thyroiditis	0.55 (0.20–1.52)	0.247	–	–
TSH	0.86 (0.65–1.13)	0.283	–	–
Nodule size	1.02 (0.99–1.05)	0.182	–	–
Solid composition	2.04 (0.74–5.66)	0.170	–	–
Hypoechoic nodule	1.53 (0.59–3.95)	0.377	–	–
Taller-than-wide shape	9.33 (2.20–39.51)	0.002	2.35 (0.36–15.25)	0.369
Irregular margin	24.06 (4.81–120.48)	<0.001	9.46 (1.88–47.59)	0.006
Presence of microcalcifications	23.40 (2.66–205.61)	0.004	2.55 (0.21–30.57)	0.460

Abbreviations: OR, odds ratio; CI, confidence interval.

**Table 7:** Receiver operating characteristic (ROC) analysis of the multivariable analysis and ACR TI-RADS for malignant histopathology.

Variable	AUC (95% CI)	Exploratory Threshold	Sensitivity (%)	Specificity (%)	*p* Value
Multivariable analysis	0.717 (0.603–0.831)	≥0.461	46.2	94.9	<0.001
Bootstrap optimism-corrected model	0.701	-	-	-	-
ACR TI-RADS category	0.704	-	-	-	-

Abbreviations: ACR, American College of Radiology; AUC, area under the receiver operating characteristic curve; CI, confidence interval; TI-RADS, Thyroid Imaging Reporting and Data System.

## Data Availability

The datasets generated and/or analyzed during the current study are not publicly available due to privacy but are available from the corresponding author upon reasonable request.

## Abbreviations

The following abbreviations are used in this manuscript:

ACR	American College of Radiology
AUC	Area Under the Receiver Operating Characteristic Curve
CI	Confidence Interval
FN/SFN	Follicular Neoplasm/Suspicious for a Follicular Neoplasm
FNA	Fine-Needle Aspiration
OR	Odds Ratio
ROC	Receiver Operating Characteristic
TSH	Thyroid-Stimulating Hormone
USG	Ultrasonography
